# Kindness Protects the Heart: The Positive Impact of Self‐Compassion on Autobiographical Memory Retrieval

**DOI:** 10.1002/brb3.71741

**Published:** 2026-09-06

**Authors:** Haihong Yin, Xiaozhuang Wang, Wei Hu, Liqing Liu, Yongmei Hu

**Affiliations:** ^1^ School of Education and Psychology Tianjin University of Sport Tianjin China; ^2^ Faculty of Psychology Tianjin Normal University Tianjin China; ^3^ Key Research Base of Humanities and Social Sciences of the Ministry of Education, Academy of Psychology and Behavior Tianjin Normal University Tianjin China; ^4^ Tianjin Social Science Laboratory of Students’ Mental Development and Learning Tianjin China

**Keywords:** autobiographical memory, brain network connectivity, event‐related potential, graph theoretical metrics, self‐compassion

## Abstract

**Objectives:**

Accumulating evidence has demonstrated the protective role of self‐compassion in mental health. However, little is known about how self‐compassion influences the cognitive and neural processes involved in autobiographical memory retrieval. The present study examined whether individuals with different levels of self‐compassion differ in the behavioral performance and neural dynamics of autobiographical memory retrieval.

**Method:**

Participants were divided into high and low self‐compassion groups according to their Self‐Compassion Scale scores. In Experiment 1, 49 participants (high = 26) completed an autobiographical memory retrieval task. In Experiment 2, 39 participants (high = 20) completed an autobiographical memory retrieval task while electroencephalography (EEG) was recorded. Event‐related potentials (ERPs) and theta‐band functional connectivity were analyzed to characterize the temporal dynamics and network properties of memory retrieval.

**Results:**

Compared with the low self‐compassion group, the high self‐compassion group showed significantly higher autobiographical memory specificity, indicating more detailed memory retrieval. ERP results showed that the low self‐compassion group exhibited a larger N1 amplitude, suggesting enhanced early attentional allocation to retrieval cues, whereas the high self‐compassion group showed a larger late positive potential (LPP), reflecting greater sustained cognitive evaluation during later stages of memory retrieval. Theta‐band network analysis further showed that the high self‐compassion group exhibited lower global efficiency and longer characteristic path length than the low self‐compassion group, indicating different patterns of functional network organization during autobiographical memory retrieval.

**Conclusion:**

These findings suggest that self‐compassion is associated with more specific autobiographical memory retrieval and distinct neural processing during both early attentional and later evaluative stages of memory retrieval, providing behavioral and neurophysiological evidence for the relationship between self‐compassion and autobiographical memory retrieval.

## Graphical abstract

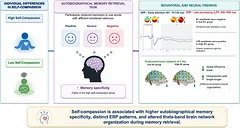

## 1 Introduction

Self‐compassion, as a core concept of positive psychological resources, was proposed by Neff (2003) based on humanistic and Buddhist theories. It encourages individuals to adopt a positive, open, and compassionate attitude toward themselves, accepting and embracing their own experiences, while acknowledging that pain and frustration are part of the shared human condition (Neff 2003; Gong et al. 2014). In recent years, a growing body of empirical research—spanning both cross‐sectional and longitudinal designs—has consistently demonstrated the protective role of self‐compassion in promoting mental health (Dai et al. 2024; Liao et al. 2021; Shin and Lim 2019). Existing research has confirmed that self‐compassion mitigates negative emotional experiences through immediate emotional regulation, thereby enhancing psychological adaptability (Wilson et al. 2019). Neurophysiological evidence indicates that self‐compassion is closely associated with brain regions involved in emotion regulation and self‐referential processing, such as the dorsolateral prefrontal cortex, insula, amygdala, and cortical midline structures. It may function by reducing excessive self‐focused immersion during the processing of negative self‐referential information (Liu et al. 2022; Liu et al. 2023; Parrish et al. 2018; Shiota 2024). Some researchers have suggested that self‐compassion facilitates the acceptance of negative experiences through self‐kindness, common humanity, and mindfulness. By reducing ruminative processing following negative events and attenuating the emotional impact of reactivated negative memories, self‐compassion may serve as a protective factor for mental health (Neff 2003; Leary et al. 2007; Blackie and Kocovski 2018; Zangri et al. 2025; Bian 2019).

Following negative life events, individuals may repeatedly recall these experiences in their subsequent lives, reexperiencing the associated emotions, reevaluating the meaning of the events, and updating their self‐perceptions during memory retrieval. Therefore, the protective role of self‐compassion may be reflected not only in individuals’ responses when negative events occur but also in how they retrieve and interpret past experiences. Previous studies have shown that self‐compassion buffers negative emotional responses when individuals recall both everyday adverse life events and severe stressful experiences, while reducing feelings of shame, self‐criticism, and psychological distress (Hiraoka et al. 2015; Johnson and O'Brien 2013; Leary et al. 2007; Miyagawa and Taniguchi 2020; Stutts et al. 2018), and has demonstrated potential value in trauma‐related interventions and psychotherapy (Valdez and Lilly 2015; Winders et al. 2020). However, existing studies have primarily relied on questionnaire surveys, retrospective reports, or single‐session behavioral assessments to examine the relationship between self‐compassion and post‐retrieval emotional experiences or overall psychological adjustment, making it difficult to elucidate how self‐compassion influences the dynamic process of autobiographical memory retrieval (Liu et al. 2023).

In studies of memory for past experiences, the Autobiographical Memory Test (AMT) has been widely used to assess individuals’ ability to retrieve autobiographical memories (Doan et al. 2025; Williams and Broadbent 1986). By providing a series of cue words, participants are asked to recall specific memories associated with them. This task can quantify indicators such as the quantity, specificity, and vividness of autobiographical memory. Autobiographical memory encompasses contextual details, emotional experiences, and subjective evaluations of personally experienced events, and plays a critical role in self‐representation, problem solving, and future behavior (Tulving 2002; Yang 1999). Impairments in autobiographical memory specificity have been closely associated with psychological disorders such as depression (Barry et al. 2021; Hallford et al. 2021). From a theoretical perspective, self‐criticism, rumination, and emotional avoidance may consume cognitive resources, thereby interfering with the search for and reconstruction of specific episodic details. In contrast, self‐compassion may reduce excessive self‐immersion and facilitate a more balanced processing of autobiographical memories. Consequently, individuals with different levels of self‐compassion may differ in the specificity and vividness of autobiographical memory retrieval. Accordingly, the present study employed the AMT to examine the association between self‐compassion and autobiographical memory retrieval performance, thereby extending our understanding of the protective role of self‐compassion in mental health from the perspective of emotion–memory interactions.

However, behavioral measures alone cannot capture the temporal dynamics underlying autobiographical memory retrieval. Autobiographical memory retrieval is, by nature, a dynamic and evolving cognitive process that involves continuous interactions among attentional resource allocation, emotional evaluation, and self‐referential processing. Therefore, shifting the focus from behavioral outcomes to the temporal dynamics of its neural mechanisms is essential for understanding its functional pathways (Liu et al. 2022; Liu et al. 2023; Shiota 2024; Stawarczyk et al. 2018). Event‐related potentials (ERPs) provide high temporal resolution, allowing neural activity to be tracked across successive stages of autobiographical memory retrieval following the presentation of retrieval cues. Autobiographical memory retrieval generally involves early stimulus identification and attentional orienting, followed by the selective processing of emotional significance, and later sustained evaluation, self‐referential processing, and cognitive resource allocation (Fields et al. 2021; Pereira et al. 2021; Tanguay et al. 2022). Based on this temporal sequence, the present study focused on the N1, early posterior negativity (EPN), and late positive potential (LPP) components. Specifically, the N1 is associated with early perceptual processing and attentional orienting, the EPN reflects relatively automatic selective processing of emotionally salient information, and the LPP is considered an index of sustained attention, emotional evaluation, and cognitive resource allocation (Amodio et al. 2014; Bauer et al. 2012; Fields et al. 2021; Luo et al. 2023; Luo et al. 2020). Examining these ERP components together enables the characterization of self‐compassion–related differences across distinct stages of autobiographical memory retrieval.

In addition, a small number of electroencephalography (EEG) studies have suggested that self‐compassion is associated with attentional allocation, emotional processing, and theta‐band activity (Creaser et al. 2022; Moadab 2013). Theta oscillations have been linked to episodic memory retrieval, cognitive control, and large‐scale neural information integration (Luo et al. 2023). However, it remains unclear at which stage of autobiographical memory retrieval self‐compassion–related differences emerge. Therefore, the present study combined ERP analysis with theta‐band functional connectivity analysis. Global efficiency and characteristic path length were used to evaluate the efficiency of information transmission and integration within functional brain networks, thereby providing network‐level evidence to complement the stage‐specific neural processes revealed by ERPs.

Therefore, this study aims to investigate how self‐compassion exerts a protective effect on mental health by regulating negative emotions during autobiographical memory retrieval, using ERP techniques to capture the temporal dynamics of this cognitive process. Additionally, we examine the brain functional connectivity patterns of individuals with varying levels of self‐compassion during memory retrieval, providing further neurocognitive evidence to elucidate the underlying mechanisms. This integrative approach offers valuable insights into the role of self‐compassion in emotional regulation and its neural correlates. This study explores the following two core questions: (1) Does self‐compassion affect autobiographical memory retrieval and its performance? (2) Through the research paradigm of autobiographical memory retrieval, explore the relationship between self‐compassion's protective advantages for mental health and negative emotion processing and cognitive processing resources.

Based on the existing literature, we proposed the following hypotheses. First, compared with the low self‐compassion group, the high self‐compassion group would exhibit greater autobiographical memory specificity and vividness. Second, compared with the high self‐compassion group, the low self‐compassion group was expected to show greater attentional sensitivity to negative retrieval cues during the early stage of memory retrieval, as reflected by a larger N1 amplitude. During the later stages of memory retrieval, the low self‐compassion group was expected to exhibit reduced sustained processing and cognitive resource allocation, as reflected by a smaller LPP amplitude. Because the EPN primarily reflects relatively automatic processing of emotionally salient information and the effects of self‐compassion are more likely to emerge during early attentional orienting and later evaluative processing, no significant group differences in EPN amplitude were expected. Differences in functional brain connectivity were examined as an exploratory analysis.

## 2 Experiment 1: The Effect of Self‐Compassion on Autobiographical Memory Retrieval

### 2.1 Research Objectives

Experiment 1 aimed to investigate whether self‐compassion influences autobiographical memory retrieval performance under different emotional valences of cue words (positive, neutral, negative), from a behavioral perspective.

### 2.2 Research Design

A 2 (group: high vs. low self‐compassion) × 3 (cue words valence: positive, neutral, negative) mixed experimental design was used. Group served as the between‐subjects variable, and cue word valence as the within‐subjects variable. The dependent variables were the specificity and vividness scores of the retrieved autobiographical memories.

### 2.3 Methods

#### 2.3.1 Participants

Based on a power analysis conducted using G^*^Power (Faul et al. 2007), a minimum of 14 participants per group (total *N* = 28) was required to detect a medium effect size (Cohen's *d* = 0.3) in a mixed‐design ANOVA, with an alpha level of 0.05 and statistical power of 0.80. The experiment was conducted by distributing the Self‐Compassion Scale (SCS) questionnaire within a university, with 194 valid responses collected. Based on the distribution of the top 27% and bottom 27% of the questionnaire total scores, participants were invited to participate in the experiment voluntarily. The inclusion criteria were as follows: participants aged 18–25 years with normal or corrected‐to‐normal vision. The exclusion criteria included (1) current use of medications that affect the central nervous system, (2) receipt of psychological counseling or psychotherapy within the past 6 months, and (3) a self‐reported history of neurological or psychiatric disorders. Ultimately, 49 participants were recruited (*M*_age_ = 19.51 years, SD = 1.50 years, age range: 18–24 years), with 26 in the high self‐compassion group and 23 in the low self‐compassion group. Before the commencement of the experiment, all participants signed a written informed consent form. The study was approved by the local ethics committee.

The self‐compassion scores of the high group were 98.92 ± 6.23, while those for the low group were 59.04 ± 8.36. An independent samples *t*‐test revealed a significant difference between the groups (*t* = 14.34, *p* < 0.001), indicating that the grouping was effective and met the experimental requirements.

#### 2.3.2 Materials

1. Chinese version of the SCS: The SCS was originally developed by Neff (2003) and consists of 26 items, comprising six subscales: self‐kindness (5), self‐judgment (5), sense of common humanity (4), isolation (4), mindfulness (4), and overidentification (4), rated on a 1–5 scale. This study uses a mature Chinese translation (Neff et al. 2017; Gong et al. 2014).
2. Autobiographical memory experimental materials: This experiment used Williams and Broadbent's (1986) AMT paradigm. Before the experiment, participants were informed of the memory retrieval requirements. A series of cue words was then presented on the screen, and participants were asked to recall related memories based on these cue words and describe them. Based on previous studies (Chen et al. 2013; Luo et al. 2020; Zhang and Xu 2011), 10 positive words, 10 negative words, and 10 neutral words were selected. The familiarity and valence of these words were reassessed, and ultimately five positive words (joy, satisfaction, courage, intelligence, and fun), five negative words (sadness, cruelty, failure, sorrow, and frustration), and five neutral words (movie, shopping mall, school, bread, and exam) were selected. There were no significant differences among the three types of words in terms of frequency, number of strokes, and familiarity.

#### 2.3.3 Experimental Procedure

**FIGURE 1:**
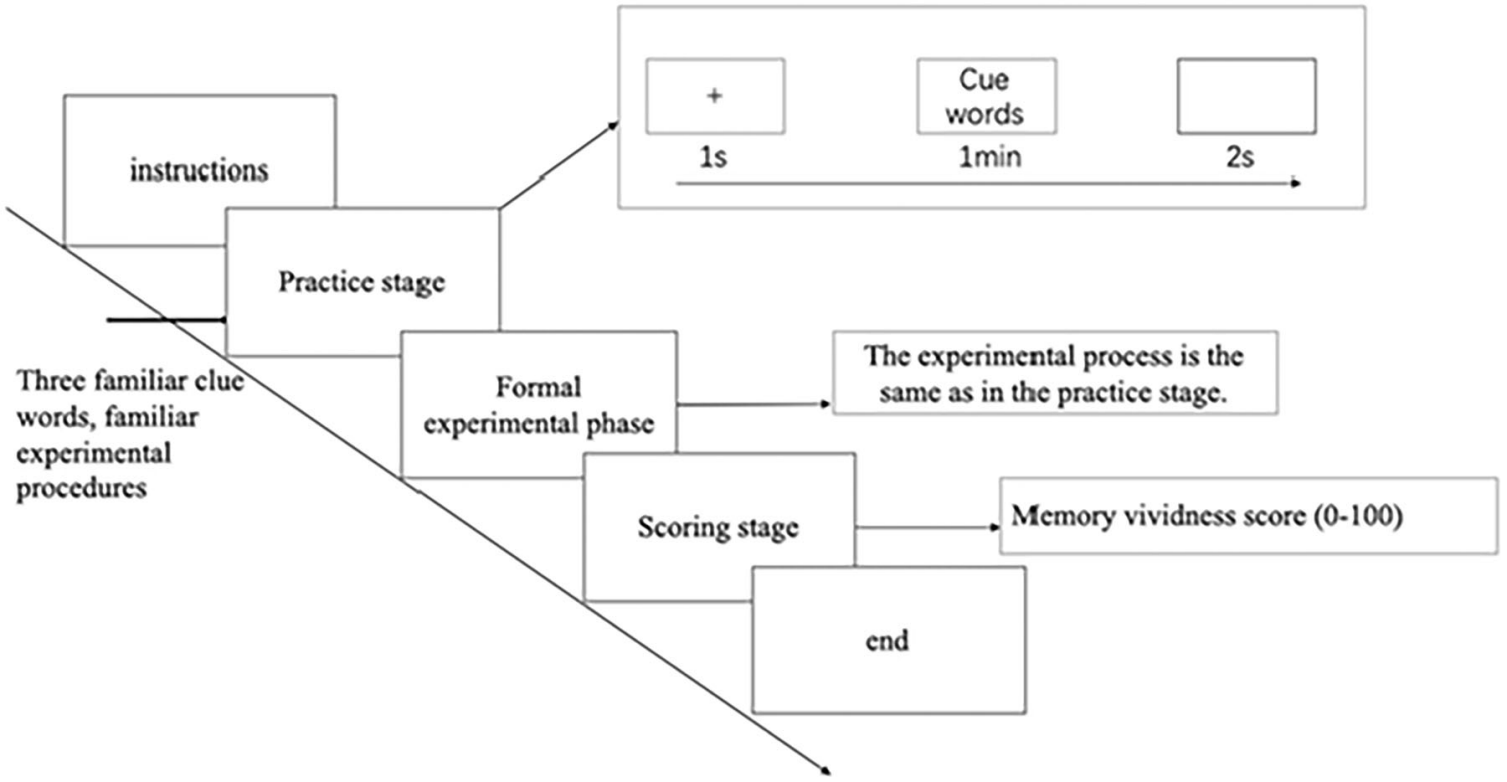
Flow chart of Experiment 1.

The experimental procedure consisted of three phases: a practice phase, a formal experimental phase, and a scoring phase. After entering the laboratory, the subjects first filled out an informed consent form (Figure 1).

1. Practice phase: Participants were shown detailed instructions on the screen to help them become familiar with the experimental process. The instructions were as follows: “In the following task, you will see a series of cue words (positive, neutral, or negative) appear on the screen. Please try to recall a personal experience related to each word and describe what you remember. Your verbal responses will be recorded and used solely for research purposes; all information will remain confidential. For example, if the word ‘beautiful’ appears, you might say: ‘Last Saturday, I went to the park with my friends. We took many beautiful photos, had a meal together, and I felt very happy.’ Please make sure to recall and describe real, specific events from your own life, rather than constructing random or fictional sentences. You will have one minute to recall and respond to each cue word.” Then, the subjects will be given three practice words to help them familiarize themselves with the specific procedures of the experiment.
2. Formal experimental phase: Cues will be presented on the screen one at a time, with each cue displayed for 1 minute. During this period, participants must recall and describe specific autobiographical memories associated with each cue until all cues have been presented.
3. Scoring phase: After completing all recall tasks, the cue words were presented again in the original experimental order. Subjects are asked to rate the vividness of each memory they retrieved. The scoring scale ranges from 0 to 100, with 0 indicating no mental imagery and 100 indicating very vivid memories with clear visual imagery.

#### 2.3.4 Data Statistics and Analysis

1. Specificity score calculation: Refer to Wenzel et al. (2004) for the evaluation method of autobiographical memory specificity. The criteria for determining specificity memory scores are as follows: A memory was awarded 1 point if it referred to a specific personal event that occurred within a single day. For example: “During winter break, I went ice skating with friends at Hongshan Park. There was a shopping mall nearby with a food court, where I bought milk tea and xiaolongbao.” This example includes clear contextual and temporal details, qualifying them as specific autobiographical events. Situations where a score of 0 is assigned include (1) the rater cannot determine whether the event occurred within a single day based on the participant's recollection, for example, “My aunt's younger brother is about to take the high school entrance exam and is staying at my house; I'm very worried about his exam”; (2) the recalled event spans more than 1 day, for example, “I've participated in many different psychology experiments over the past month and found them quite interesting”; (3) the event recalled is a routine, recurring occurrence in daily life, for example, “Every Wednesday, I go to a buffet”; (4) the participant recalls an event that is not a personal experience but rather an imagination or wish about the present or future, such as: “Next week is exam week, and I feel anxious. I hope to pass smoothly”; (5) the participant creates a sentence or explanation for the cue word, such as: “When I see ‘smart,’ I think of big ears, and I think people with big ears are smart.”
2. Vividness score calculation method: The vividness score is calculated as the average of the self‐rated scores given by the subjects for the vividness of the retrieved memory content in each task.

All data were analyzed using SPSS version 26.0. A 2 (group: high vs. low self‐compassion) × 3 (cue word valence: positive, neutral, negative) mixed‐design repeated measures ANOVA was conducted to examine the effects on both specificity and vividness scores of autobiographical memories.

### 2.4 Research Results

#### 2.4.1 Specificity Scores

**FIGURE 2:**
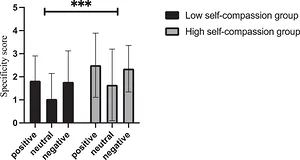
Self‐compassion influences the specificity score of autobiographical memory.

A repeated‐measures analysis of variance (ANOVA) was conducted with specificity scores as the dependent variable and group (high vs. low self‐compassion) and cue word valence (positive, neutral, negative) as independent variables (Figure 2). The results indicated that the main effect of group was significant, *F*(1, 47) = 6.81, *p* = 0.012, *η* ^2^_p_ = 0.13. The autobiographical memory specificity scores of the high group (*M*_low_ = 1.55, SD_low_ = 0.172) were significantly higher than those of the low group (*M*_high_ = 2.17, SD_high_ = 0.162, 95% CI = [−1.09, 0.14]). The main effect of the emotional valence of cue words was significant, *F*(2, 94) = 6.94, *p* = 0.002, *η* ^2^_p_ = 0.13. The scores for autobiographical memory specificity induced by positive cue words (*M*_positive_ = 2.16, SD_positive_ = 0.18) were higher than those for neutral cue words (*M*_neutral_ = 1.35, SD_neutral_ = 0.20, 95% CI = [−1.80, 2.52]), while the specificity scores of autobiographical memory guided by negative cue words (*M*_Negative_ = 2.06, SD_Negative_ = 0.17) were higher than those guided by neutral cue words (*M*_Neutral_ = 1.35, SD_Neutral_ = 0.20, 95% CI = [0.22, 1.26]). The interaction between group and cue word valence was not significant, *F*(2, 94) = 0.03, *p* = 0.97.

#### 2.4.2 Vividness Score

**FIGURE 3:**
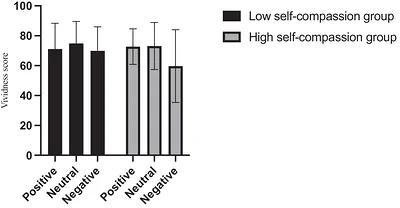
Self‐compassion influences vividness scores of autobiographical memories.

A repeated measures ANOVA was conducted with the vividness score of autobiographical memory as the dependent variable (Figure 3). The results showed that the main effect of group was not significant, *F*(1, 47) = 0.89, *p* = 0.35, *η* ^2^_p_ = 0.02 (*M*_high_ = 71.97, SD_high_ = 2.67, SD_low_ = 2.51, *M*_low_ = 68.51, 95% CI = [−3.91, 10.85]). The main effect of cue word valence was significant, *F*(2, 94) = 5.85, *p* = 0.007, *η* ^2^_p_ = 0.11. The vividness of negative autobiographical memory (*M*_Negative_ = 64.80, SD_Negative_ = 2.98) was significantly lower than that of neutral words (*M*_Neutral_ = 74.01, SD_Neutral_ = 2.18, *p* < 0.001, 95% CI = [−14.36, −4.07]), and also lower than the vividness of autobiographical memory for positive cue words (*M*_Positive_ = 71.90, SD_Positive_ = 2.10, *p* = 0.045, 95% CI = [0.17, 14.03]). The interaction between group and autobiographical memory affect valence was not significant, *F*(2, 94) = 2.31, *p* = 0.12, *η* ^2^_p_ = 0.05.

### 2.5 Discussion

The purpose of Experiment 1 was to explore whether self‐compassion affects individuals' performance in autobiographical memory retrieval tasks, focusing on the specificity and vividness of memory. It was hypothesized that individuals with higher levels of self‐compassion would retrieve more specific and vivid autobiographical memories, reflecting their advantages in emotional regulation and potentially indicating more efficient cognitive processing during memory retrieval.

In terms of specificity, the results showed that the high self‐compassion group scored significantly higher than the low self‐compassion group on the specificity of autobiographical memory, which was consistent with the hypothesis. These findings suggest that individuals with higher levels of self‐compassion are more likely to retrieve autobiographical memories with clearly defined temporal, spatial, and event‐specific details. According to the conceptual framework of self‐compassion, individuals with higher self‐compassion tend to approach self‐relevant experiences with self‐kindness, a sense of common humanity, and mindfulness, exhibiting less self‐criticism and overidentification with negative thoughts and emotions (Neff 2003, 2023). From the perspective of the Self‐Memory System, the retrieval of specific autobiographical memories requires a progressive search from general self‐knowledge to event‐specific knowledge (Conway and Pleydell‐Pearce 2000). Accordingly, individuals with higher self‐compassion may engage in memory search in a more open and accepting manner, making them less likely to terminate the search process prematurely at the level of general autobiographical knowledge because of self‐criticism or emotional discomfort, and therefore more likely to retrieve event‐specific information. The low self‐compassion group had lower specificity, possibly to avoid negative emotional experiences, thereby compressing the specific details of the memory. Compared to more vivid and specific memory retrieval, vague and generalized recollections do not trigger strong emotional distress, which helps alleviate individuals' pain associated with past events (Ahmadi Forooshani et al. 2020).

In terms of memory vividness scores, the main effect of group was not significant. One possible explanation is that self‐reported vividness may be more vulnerable to fluctuations in individuals’ current emotional states, task engagement, or subjective evaluation criteria (Berntsen and Rubin 2008; Li and Zhao 2016). Although individuals with high self‐compassion may have advantages in cognitive processing, in subsequent scoring sessions without pressure or emotional triggers, individuals may not necessarily mobilize more psychological resources to enhance the detailed processing of memory. Therefore, no differences were observed in the vividness scores after the experiment.

In addition, the study also found that the emotional valence of cue words has a significant impact on the vividness and specificity of memory, with positive and neutral cues eliciting memories that are more vivid and specific than negative cues. This suggests that emotional cues play a critical role in facilitating memory retrieval, with positive and neutral cues more likely to activate detailed memory traces. Conversely, under negative emotional cues, individuals may tend to avoid or suppress emotionally distressing content, which in turn reduces the specificity and vividness of the retrieved memories (Conway and Pleydell‐Pearce 2000; Gong et al. 2019).

Experiment 1 revealed an overall behavioral difference in autobiographical memory specificity between individuals with high and low levels of self‐compassion. However, behavioral measures alone could not identify the processing stage at which this difference emerged. Therefore, Experiment 2 employed ERP techniques to characterize the temporal dynamics of autobiographical memory retrieval, providing complementary neural evidence for the behavioral findings of Experiment 1.

## 3 Experiment 2: The Effect of Self‐Compassion on Brain Activity During Autobiographical Memory Retrieval

### 3.1 Research Objectives

Study 2 aims to investigate the neural mechanisms underlying the influence of self‐compassion on autobiographical memory retrieval using ERP technology. Specifically, this study will examine differences in behavioral response times and ERP components between high and low self‐compassion groups, focusing on the following three components: N1 amplitude, an indicator of early visual attention, reflecting the distribution of attention at the initial stage of memory retrieval. EPN amplitude (early negative component), related to the automatic processing of emotional stimuli, represents the rapid detection and early processing of emotional information. LPP amplitude (late positive component) reflects the sustained processing and cognitive evaluation of emotional stimuli, representing the delayed response and deep processing of emotional memories by individuals. Furthermore, this study will analyze brain network differences between the two groups based on functional connectivity to explore whether and how self‐compassion modulates autobiographical memory retrieval through the regulation of neural network activity. By examining these electrophysiological markers and neural connectivity patterns, this study aims to elucidate the temporal dynamics and cognitive processes through which self‐compassion exerts its effects during autobiographical memory retrieval.

### 3.2 Methods

#### 3.2.1 Participants

Statistical power analysis was conducted using G^*^Power software, similar to Study 1. The SCS was administered via an online questionnaire. Participants were selected based on the top 27% and bottom 27% of total scores from the validly returned questionnaires, resulting in high and low self‐compassion groups, respectively. Following the principle of voluntary participation, a total of 44 undergraduate students from a general university were recruited. The inclusion criteria were as follows: participants aged 18–25 years with normal or corrected‐to‐normal vision. The exclusion criteria included (1) current use of medications that affect the central nervous system, (2) receipt of psychological counseling or psychotherapy within the past 6 months, and (3) a self‐reported history of neurological or psychiatric disorders. Among them, four participants had severe EEG amplitude drift, resulting in insufficient valid trials, and one participant did not complete the experiment; their data were excluded. Ultimately, data from 39 participants were included in the statistical analysis (*M*_age_ = 20.92 years, SD = 12.03 years, range: 18–24 years), with 20 in the high self‐compassion group and 19 in the low self‐compassion group (10 males, 5 in each group). All participants had normal vision and corrected vision; none had participated in similar experiments before. All participants signed informed consent forms before the experiment began, and the experimental procedure was approved by the local ethics committee.

#### 3.2.2 Research Design

A two‐factor mixed experimental design was employed, consisting of a 3 (autobiographical memory retrieval cue valence: positive, neutral, negative) × 2 (group: high self‐compassion, low self‐compassion) structure. Cue valence was treated as a within‐subjects variable, and self‐compassion group as a between‐subjects variable. And the dependent variables were the number of autobiographical memories retrieved and vividness scores; brainwave data indicators such as N1, EPN, and LPP average amplitude.

#### 3.2.3 Research Materials

1. Self‐Compassion Questionnaire: Same as Study 1
2. Paradigm: The paradigm was adapted from the autobiographical memory retrieval tasks developed by Bauer et al. (2012) and Maki et al. (2013). A total of 33 neutral nouns were used as memory cue words, with emojis guiding memory type—A smiling face emoji signaled the retrieval of a positive memory, a crying face indicated a negative memory, and a neutral face represented a neutral memory. All cue words were matched for stroke count, word frequency, and familiarity, with no significant differences across the three valence conditions.
3. Experimental program writing: E‐Prime 3.0 was used for writing. The experiment was divided into six blocks, each containing five trials, with each block repeated six times, for a total of 180 trials.

#### 3.2.4 Experimental Procedure

**FIGURE 4:**
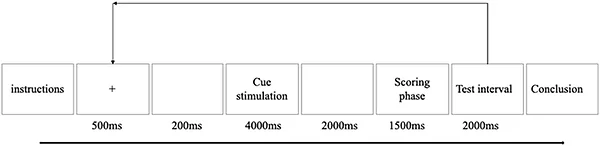
Flowchart of Experiment 2.

Upon entering the laboratory, participants read and signed the informed consent form for participants. Following this, all necessary preparations for EEG data acquisition were completed. Participants were then fitted with an electrode cap and seated individually in a dimly lit, electrically shielded room. The eyes are approximately 70 cm away from the computer screen. The stimulus is presented at the center of the screen, with black text on a white background, using 40‐point Song font. For each trial, a 500‐ms “+” fixation point is first presented to draw attention, followed by a 200‐ms blank screen. Then, the experimental stimulus is presented for 4000 ms, followed by a 2000‐ms blank screen. The scoring phase then begins, lasting 1500 ms, followed by another 2000‐ms blank screen. The participants' task was to view the stimulus material and retrieve memories that met the specified criteria. If they recalled a memory, they pressed the “p” key; if they could not recall any memories, they did not press any key. During the scoring phase, if the recalled memory is vivid and vivid, as if it had just happened, press “2”; if the memory is somewhat vague, press “1”; if no memory is retrieved, press “0.” A rest period is set between blocks, and after the rest period, the next block begins. The detailed process is as follows (Figure 4).

#### 3.2.5 EEG Recording and Analysis

1. Data preprocessing: EEG data were recorded using a BrainAmp DC amplifier (Brain Products, Germany) with a 64‐channel electrode cap arranged according to the international 10–20 system. The electrode impedance was less than 5 kΩ, and the sampling rate was 500 Hz/channel. During online recording, the bilateral mastoid points were used as reference electrodes, and the filter bandwidth was set to 0.1–30 Hz. The baseline was set at 200 ms for the presentation of memory cue words, and the EEG analysis time window was set at 1500 ms. Following the method of Gratton et al. (1983) for removing eye movement artifacts, artifacts exceeding ±100 µV were excluded. Only successfully retrieved memories were included in the ERP analyses. Participants with fewer than five artifact‐free epochs in any condition were excluded. The final sample consisted of 39 participants, all of whom retained substantially more valid trials than the exclusion criterion. Across the three experimental conditions, the mean numbers of retained trials were 51.69, 51.44, and 51.13, respectively, yielding an overall trial retention rate of 85.70%.
2. Data analysis: Based on the research objectives and relevant prior studies (Bauer et al. 2012; Lu et al. 2011; Luo et al. 2020), the following ERP components were selected for analysis: N1 (70–130 ms; electrodes FC1, FC2, FCz), EPN (150–300 ms; electrodes P3, P4, P7, P8, O1, O2), and LPP (400–700 ms, 700–1000 ms, and 1000–1500 ms; electrodes F1, F2, Cz). A repeated measures ANOVA was conducted on the average amplitudes under different cue valence conditions. Specifically:

The N1 component was defined as a negative deflection occurring within the 70–130 ms time window and is considered to primarily reflect early automatic attentional processing. This component is typically most prominent in the fronto–central regions (FC1, FC2, and FCz) (Lu et al. 2011; Foti et al. 2009). Accordingly, these electrode sites were selected as the regions of interest (ROIs) for the N1 analysis.

The EPN component was defined as a negative‐going waveform occurring within the 150–300 ms time window and is associated with prioritized emotional processing. It is generally most pronounced in temporo‐occipital regions (P3, P4, P7, P8, O1, and O2) and is closely related to the emotional arousal level of stimuli (Bauer et al. 2012; Hajcak and Dennis 2009). Therefore, these electrode sites were selected for the EPN analysis.

The LPP component typically emerges after 400 ms and may persist into later stages of cognitive evaluation. In the present study, the LPP was further divided into three time windows (400–700 ms, 700–1000 ms, and 1000–1500 ms) to more precisely characterize sustained cognitive resource allocation during late‐stage processing (Luo et al. 2023; Luo et al. 2020). The LPP is generally most prominent in midline and fronto‐central regions (F1, F2, and Cz); therefore, these electrode sites were selected for analysis.

When the assumption of sphericity was violated, the Greenhouse–Geisser correction was applied to adjust the *p*‐values.

1. Functional connectivity: Based on previous studies, after preliminary preprocessing of the data, segmented EEG data were obtained. Since the brain is a nonlinear dynamical system, calculating the degree of cooperation between different brain regions using amplitude‐based methods is easily affected by noise (Li et al. 2019). In contrast, phase‐based phase locking value (PLV) is highly sensitive to synchronous changes between different phases. Therefore, this study selected the PLV as the indicator for calculating the correlation between two leads. The formula is as follows:
$$\mathrm{PLV}=\frac{1}{N}\left|\Sigma_{\left(n=1\right)}^{N}\exp\left(i\theta \left(t\right)\right)\right|$$

The PLV ranges from 0 to 1. The higher the PLV between two nodes, the stronger the correlation between the brain regions corresponding to the two nodes. Each lead is a node, and the strength of the PLV is the edge. Using this method, we obtained an adjacency matrix based on EEG channel signals, constructed a brain network based on functional connectivity, and performed a comparative analysis of the networks to compare the differences between the two conditions.

1. Graph theory analysis: Graphs (or networks) are mathematical models commonly used to characterize complex systems, including the brain. In the context of brain network analysis, efficiency refers to the effectiveness of information transfer between brain regions (Pan et al. 2018). In the present study, functional connectivity matrices (60 × 60) were constructed using PLVs, and graph‐theoretical analyses were conducted using the Brain Connectivity Toolbox (BCT) in MATLAB.

To construct binary undirected graphs, PLV‐based correlation matrices were first thresholded to generate adjacency matrices. Graph metrics such as clustering coefficient and shortest path length were then computed to assess network characteristics (Li et al. 2015). Threshold values ranging from 0.1 to 0.9 (with a step size of 0.1) were tested to determine appropriate sparsity levels. It was observed that at thresholds of 0.2, 0.3, and 0.4, the resulting network sparsity was below 50% and above the minimum sparsity threshold defined as 2ln*N* **/** *N*, to retain as many network connections as possible for analysis (Erdos and Rényi 1960), while avoiding network fragmentation caused by excessive sparsity, the present study restricted the sparsity range to 0.2–0.4 under the condition that network connectivity was preserved. This range is also consistent with the sparsity thresholds commonly adopted in previous functional brain network studies (Achard and Bullmore 2007; Van Wijk et al. 2010). In subsequent analyses, to maximize the preservation of network structural information while ensuring comparability across participants, a sparsity threshold of 0.2 that satisfied both connectivity and stability criteria was selected for the construction of binary networks (Bordier et al. 2017; Van Wijk et al. 2010; Rubinov and Sporns 2010).

### 3.3 Results Analysis

#### 3.3.1 Behavioral Data

1. Number of autobiographical memory retrievals

The number of autobiographical memories retrieved served as an indicator of memory specificity. A repeated measures ANOVA was conducted with this variable as the dependent measure. The main effect of group was not significant, *F*(1, 37) = 0.66, *p* = 0.42, *η* ^2^_p_ = 0.02, 95% CI = [−0.7, 0.3]. The main effect of memory retrieval cues was not significant, *F*(2, 74) = 0.79, *p* = 0.46, *η* ^2^_p_ = 0.02, 95% CI = [−0.7, 0.3]. The interaction between group and memory retrieval cues showed a significant margin, *F*(2, 74) = 2.61, *p* = 0.08, *η* ^2^_p_ = 0.07. Simple effects analysis revealed that in negative autobiographical memory, the number of memories retrieved by the low self‐compassion group (*M*_low_ = 9.05, SD_low_ = 0.24) was significantly lower than that of the high self‐compassion group (*M*_high_ = 9.75, SD_high_ = 0.24), *F*(1, 37) = 4.31, *p* = 0.045, *η* ^2^_p_ = 0.10, 95% CI = [−1.38, −0.02].

1. Vividness of autobiographical memory retrieval

Using the vividness of autobiographical memory retrieval as the dependent variable, a 3 (autobiographical memory retrieval cues: positive, negative, neutral) × 2 (group: high self‐compassion group, low self‐compassion group) repeated measures ANOVA revealed a significant main effect of group, *F*(1, 37) = 6.34, *p* = 0.012, *η* ^2^_p_ = 0.013. The vividness scores of the low self‐compassion group (*M*_low_ = 1.66, SD_low_ = 0.01) were significantly lower than those of the high self‐compassion group (*M*_high_ = 1.71, SD_high_ = 0.01, 95% CI = [−0.1, −0.01]). The main effect of autobiographical memory retrieval cues was significant, *F*(2, 74) = 113.17, *p* < 0.001, *η* ^2^_p_ = 0.195. Pairwise comparisons revealed that the vividness of positive autobiographical memories (*M*_positive_ = 1.79, SD_positive_ = 0.01) was significantly greater than that of negative autobiographical memories (*M*_negative_ = 1.56, SD_negative_ = 0.02, *p* < 0.001, 95% CI = [0.20, 0.26]). The vividness of neutral autobiographical memories (*M*_Neutral_ = 1.70, SD_Neutral_ = 0.02) was significantly greater than that of negative autobiographical memories (*M*_Negative_ = 1.56, SD_Negative_ = 0.02), *p* < 0.001, 95% CI = [0.11, 0.17]. The interaction between group and autobiographical memory retrieval type was not significant, *F*(2, 74) = 0.22, *p* = 0.81, *η* ^2^_p_ < 0.01.

#### 3.3.2 EEG Results

**FIGURE 5:**
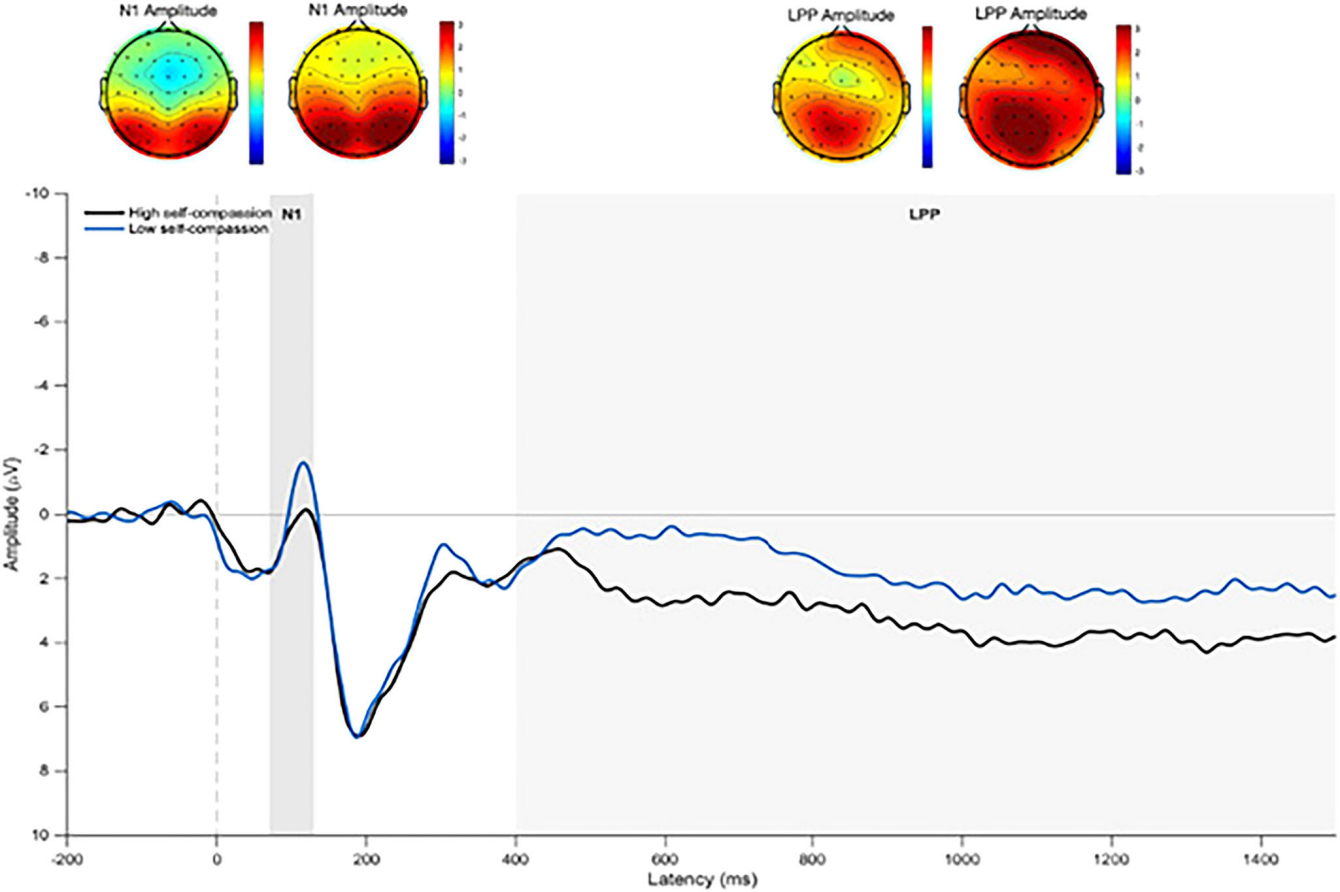
Left: waveform diagram of the low group at the CZ point; right: waveform diagram of the high self‐compassion group at the CZ point.

The analysis of EEG indicators revealed that there were significant differences between the groups, mainly in the N1 and LPP components, while no significant effect of group was found in the EPN amplitude. Figure 5 shows the EEG waveforms of the high and low self‐compassion groups.

In the average amplitude of N1 (FC1, FC2, FCZ), repeated measures ANOVA revealed a significant main effect of group, *F*(1, 37) = 24.85, *p* < 0.001, *η* ^2^_p_ = 0.18. Compared with the high self‐compassion group (*M*_high_ = 0.82, SD_high_ = 0.27), the low self‐compassion group (*M*_low_ = −1.17, SD_low_ = 0.29) exhibited more negative N1 amplitude (*p* < 0.001, 95% CI = [−2.78, −1.20]); the main effect of autobiographical memory retrieval cues was not significant, *F*(2, 74) = 0.42, *p* = 0.63, *η* ^2^_p_ = 0.004; the interaction between group and autobiographical memory retrieval cue was significant, *F*(2, 74) = 4.07, *p* = 0.022, *η* ^2^_p_ = 0.034. Post hoc simple effects analysis revealed that in the low self‐compassion group, the average N1 amplitude during negative autobiographical memory retrieval (*M*_Negative_ = −1.55, SD_Negative_ = 0.39) was smaller than that during positive autobiographical memory retrieval (*M*_Positive_ = −0.99, SD_Positive_ = 0.30, *p* = 0.056, 95% CI = [−0.01, 1.14]); whereas in the high self‐compassion group, the average N1 amplitude during the retrieval of negative autobiographical memories (*M*_Negative_ = 1.12, SD_Negative_ = 0.38) was greater than that during the retrieval of positive autobiographical memories (*M*_Positive_ = 0.50, SD_Positive_ = 0.29, *p* = 0.033, 95% CI = [−1.17, −0.05]).

On the average amplitude of EPN (P3, P4, P7, P8, O1, O2), repeated measures ANOVA revealed that the main effect of group was not significant, *F*(1, 37) = 1.21, *p* = 0.27, *η* ^2^_p_ = 0.005, 95% CI = [−0.40, 1.43]; the main effect of autobiographical memory retrieval cues was significant, *F*(2, 74) = 3.60, *p* = 0.030, *η* ^2^_p_ = 0.015. The mean amplitude of positive autobiographical memories (*M*_positive_ = 5.10, SD_positive_ = 0.25) was greater than that of neutral memories (*M*_neutral_ = 4.82, SD_neutral_ = 0.24, *p* = 0.009, 95% CI = [0.07, 0.50]), and negative (*M*_Negative_ = 4.81, SD_Negative_ = 0.24, *p* = 0.024, 95% CI = [0.04, 0.54]); the interaction between group and autobiographical memory retrieval type was not significant, *F*(2, 74) = 1.37, *p* = 0.25, *η* ^2^_p_ = 0.006.

Repeated measures ANOVA was conducted on the average amplitude of LPP (F1, F2, CZ), with the following results:

At 400–700 ms, the main effect of group was not significant, *F*(1, 37) = 0.87, *p* = 0.35, *η* ^2^_p_ = 0.007; the main effect of autobiographical memory retrieval cue was significant, *F*(2, 74) = 3.20, *p* = 0.047, *η* ^2^_p_ = 0.027. The mean amplitude of positive autobiographical memories (*M*_positive_ = 2.08, SD_positive_ = 0.33) was smaller than that of neutral memories (*M*_neutral_ = 2.60, SD_neutral_ = 0.36, *p* = 0.039, 95% CI = [−0.95, −0.03]), the mean amplitude of negative autobiographical memories (*M*_Negative_ = 1.93, SD_Negative_ = 0.39) was smaller than that of neutral memories (*M*_Neutral_ = 2.60, SD_Neutral_ = 0.36, *p* = 0.034, 95% CI = [0.05, 1.24]; the interaction between group and autobiographical memory retrieval type was significant, *F*(2, 74) = 3.36, *p* = 0.040, *η* ^2^_p_ = 0.028. Simple effects analysis revealed that, under negative cue‐word conditions, the high self‐compassion group showed larger mean autobiographical memory amplitudes (*M*_high_ = 2.62, SD_high_ = 0.54) than the low self‐compassion group (*M*_low_ = 1.23, SD_low_ = 0.55, *p* = 0.075, 95% CI = [−0.14, 2.91]). No significant group difference was observed under positive cue‐word conditions. For 700–1000 ms: Only the main effect of autobiographical memory retrieval cues was significant, *F*(2, 74) = 3.49, *p* = 0.035, *η* ^2^_p_ = 0.029.

For 1000–1500 ms: The main effect of group was significant, *F*(1, 37) = 4.23, *p* = 0.042, *η* ^2^_p_ = 0.035. The mean amplitude of the low self‐compassion group (*M*_low_ = 2.57, SD_low_ = 0.47) was smaller than that of the high self‐compassion group (*M*_high_ = 3.92, SD_high_ = 0.46, *p* = 0.042, 95% CI = [−2.66, −0.05]); the main effect of autobiographical memory retrieval cues was marginally significant, *F*(2, 74) = 2.59, *p* = 0.082, *η* ^2^_p_ = 0.022; the interaction between group and autobiographical memory retrieval type was significant, *F*(2, 74) = 3.15, *p* = 0.049, *η* ^2^_p_ = 0.027. Simple effects analysis revealed that under negative cue words, the mean amplitude of the high group's autobiographical memory (*M*_high group_ = 4.31, SD_high group_ = 0.51) was significantly greater than that of the low group (*M*_low group_ = 2.25, SD_low group_ = 0.52, *p* = 0.005, 95% CI = [−3.50, −0.62]), while under positive cue words, the difference was not significant.

1. Brain network connectivity results

A 60 × 60 functional connectivity matrix was constructed for each participant, yielding a total of 3540 PLV indices. Functional connectivity in the theta band was calculated between brain regions associated with autobiographical memory retrieval for both the high self‐compassion group and the low self‐compassion group. Similarities and differences in electrode‐level connectivity patterns during the task were compared between the two groups. All results were corrected using the false discovery rate (FDR) method, and only connections with significance levels of *p* < 0.001 were retained for analysis, as presented in Table 1.

**TABLE 1:** Electrode pairs showing significant differences after FDR correction.

Frequency band	Electrode pairs, high group < low group	*p*‐value after FDR correction	Corresponding brain region
Theta(4–7 Hz)	FC1–C4	< 0.001	Forehead–central back
Alpha1(8–10 Hz)	TP8–FT7	< 0.001	Temporal lobe
Alpha2(10–12 Hz)	PZ–F4	< 0.001	Precentral gyrus–middle frontal gyrus
Beta1(13–20 Hz)	TP8–CP5	< 0.001	Temporal lobe–superior temporal gyrus
Beta2(20–30 Hz)	P1–PO3	< 0.001	Precentral gyrus–occipital lobe

**FIGURE 6:**
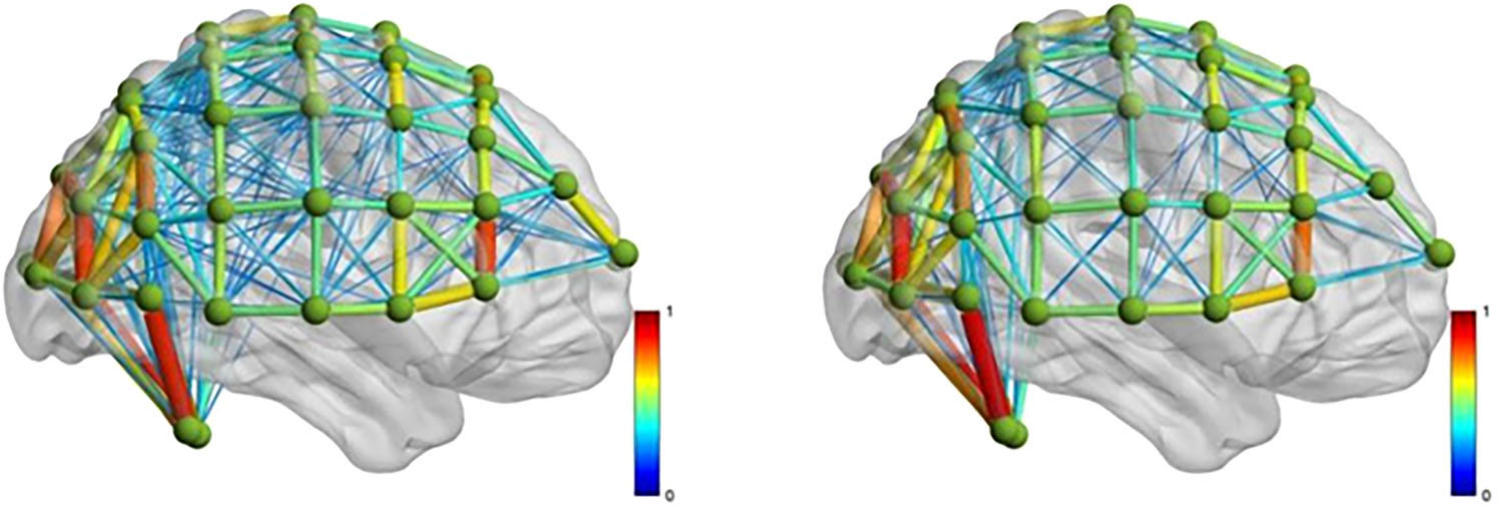
Theta‐band functional brain network connectivity based on phase‐locking value (PLV) during autobiographical memory retrieval in the low and high self‐compassion groups. The left panel represents the low self‐compassion group, and the right panel represents the high self‐compassion group. Nodes represent EEG electrodes, and colored lines indicate significant PLV‐based functional connections between electrode pairs, with warmer colors representing stronger connectivity.

To more clearly visualize the functional connectivity of brain regions during autobiographical memory retrieval in the high and low self‐compassion groups, BrainNet Viewer was used to generate brain network maps. The results indicated distinct patterns of brain network connectivity between the two groups during autobiographical memory retrieval. Figure 6 illustrates the theta‐band brain networks for both the high and low self‐compassion groups.

**FIGURE 7:**
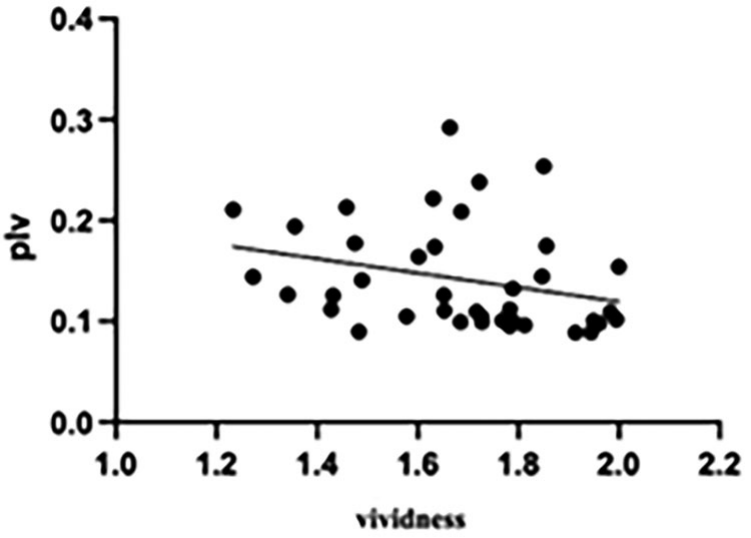
Correlation between electrode pairs with significant differences in the theta band and vividness scores.

A correlation analysis was conducted between the PLV values of electrode pairs that showed significant differences in the theta band and the participants’ average memory vividness scores across the three cue conditions. The results indicated a negative correlation (*r* = −0.28, *p* = 0.084), suggesting a trend‐level association between increased neural synchrony and reduced subjective vividness of autobiographical memory. Figure 7 presents the scatter plot illustrating this relationship.

Graph theory parameters: Graph‐theoretical analysis was performed on the brain functional connectivity networks of the high and low self‐compassion groups across different frequency bands, focusing on clustering coefficient, global efficiency, local efficiency, and characteristic path length. Global efficiency reflects the overall efficiency of information integration across the brain network, whereas characteristic path length represents the average shortest path required for information transmission between nodes. Lower global efficiency and a longer characteristic path length generally indicate reduced overall information integration efficiency within the brain network. In the theta frequency band, significant group differences were observed in global efficiency and characteristic path length. Specifically, the high self‐compassion group exhibited lower global efficiency and longer characteristic path length compared to the low self‐compassion group (Table 2).

**TABLE 2:** Results of independent sample *t*‐tests for brain network theory parameters in the high and low self‐compassion groups performing autobiographical memory retrieval tasks in the theta band.

	Degree of freedom	*t*	*p*
Global efficiency	37	2.12	0.04
Feature path length	37	−2.26	0.03

### 3.4 Discussion

Study 2 further utilized ERP and brain functional connectivity analysis methods to explore the neural processing mechanisms of self‐compassion levels in autobiographical memory retrieval. Differences between individuals with high and low self‐compassion in completing autobiographical memory tasks were examined at three levels: behavior, ERP components, and brain networks. The aim was to reveal the potential mechanisms of self‐compassion as a psychological protective factor in emotional memory processing and cognitive resource allocation from multiple dimensions.

At the behavioral level, consistent with the results of Study 1, in terms of specificity, the low self‐compassion group showed significantly fewer negative memory retrievals than the high self‐compassion group, validating the hypothesis of Study 1; in terms of memory vividness, the low self‐compassion group scored significantly lower on vividness than the high self‐compassion group, supporting the influence of self‐compassion on the quality of memory retrieval. ERP results further revealed that, compared to the high self‐compassion group, the low self‐compassion group exhibited more negative N1 wave amplitudes in the early stages and smaller LPP wave amplitudes in the middle and late stages of the stimulus. This result supports the research hypothesis, indicating that lower self‐compassion levels are associated with more negative emotional processing and more difficult memory retrieval processes.

N1 is a negative ERP component typically distributed over the mid‐parietal scalp regions. During the processing of visual stimuli, N1 amplitudes tend to be larger in response to threatening stimuli than to pleasant ones, reflecting an attentional bias (Sun et al. 2012). In this study, the low self‐compassion group exhibited significantly larger negative N1 amplitudes, suggesting greater sensitivity and heightened attentional allocation to stimulus cue words compared to the high self‐compassion group. Furthermore, the high self‐compassion group showed greater average N1 amplitudes when retrieving positive autobiographical memories relative to neutral and negative ones, while the low self‐compassion group displayed the opposite pattern. These results indicate an attentional bias toward negative stimuli in the low self‐compassion group during task processing, whereas the high self‐compassion group demonstrated an attentional preference for positive stimuli (Bian 2019).

During the middle and late stages of autobiographical memory retrieval, the LPP amplitude induced in the low self‐compassion group was smaller than that in the high self‐compassion group. The LPP component is closely related to the memory formation process, and its amplitude is determined by the allocation of psychological resources. In emotion processing research, the LPP component is regarded as an indicator reflecting analytical evaluation processes, as these processes consume significant psychological resources. Stimuli that are thoroughly analyzed and evaluated will elicit larger LPP amplitudes (Huang and Luo 2009). During the middle and late stages of autobiographical memory retrieval, the LPP amplitude of the low self‐compassion group was smaller than that of the high self‐compassion group, indicating that the low self‐compassion group invested fewer psychological resources in the autobiographical memory retrieval process. Correspondingly, in terms of behavior, the low self‐compassion group showed lower specificity and vividness in autobiographical memory retrieval.

In the functional connectivity results, the low self‐compassion group exhibited a more complex connectivity pattern than the high self‐compassion group. Significant differences were primarily observed between electrodes over frontal, central, temporal, and occipital scalp regions, suggesting that the low self‐compassion group may show greater neural coordination across these scalp regions. The integration and efficiency of information processing within brain functional networks are typically evaluated using two key metrics: characteristic path length (L) and global efficiency. Characteristic path length reflects the average shortest path between any two nodes in the network, with shorter path lengths indicating stronger functional integration and more direct interregional communication. Global efficiency, defined as the average inverse of the shortest path length, is used to quantify the overall efficiency of information transfer and processing within the network (Li et al. 2019).

In the theta band, the low self‐compassion group exhibited significantly shorter feature path lengths than the high self‐compassion group during the autobiographical memory retrieval task, while global efficiency showed the opposite pattern. This may suggest that the low self‐compassion group shortened feature path lengths by enhancing direct connections between distant brain regions during the task. However, this network characteristic may also reflect a tendency toward network randomization, implying that their cognitive processing capabilities may be somewhat limited. Previous studies on patients with depression support this view, finding that their brain networks exhibit randomized network characteristics of reduced feature path length and increased global efficiency in the δ and θ bands (Leistedt et al. 2009). Randomized networks typically feature lower modular information processing capacity and fault tolerance, which may be associated with abnormal changes in brain network hub functions (Zhang et al. 2011). Further analysis revealed a negative correlation between the vividness scores of autobiographical memory retrieval and the PLV between electrode pairs showing significant differences in the theta band. This result suggests that when memory retrieval vividness is higher and more cognitive processing resources are invested, functional connectivity between certain electrode pairs in the theta band associated with memory processing may weaken.

## 4 General Discussion

This study employed an autobiographical memory retrieval task paradigm in combination with ERP techniques to systematically investigate the influence of self‐compassion on autobiographical memory retrieval, examining both behavioral performance and neural processing. Across two studies, the protective role of self‐compassion was demonstrated from distinct perspectives. Study 1 focused on behavioral outcomes, showing that individuals with high self‐compassion exhibited greater specificity in memory retrieval. Study 2 integrated ERP and brain network analyses, uncovering neural mechanisms related to early attentional monitoring, later‐stage cognitive resource allocation, and modular network organization. Compared to the low self‐compassion group, the high self‐compassion group demonstrated superior specificity in autobiographical memory retrieval and was more capable of allocating cognitive resources for the effective retrieval and processing of emotional memories.

### 4.1 The High Self‐Compassion Group Demonstrated Superior Emotional Autobiographical Memory Retrieval Ability

The findings from both Study 1 and Study 2 consistently indicate that individuals with high self‐compassion demonstrate greater vividness during autobiographical memory retrieval tasks, suggesting a close link between self‐compassion and higher‐quality self‐narrative processing. This may be attributed to elevated levels of self‐kindness, mindfulness, and a sense of common humanity among high self‐compassion individuals (Neff 2003). Such individuals tend to exhibit greater self‐acceptance and a more adaptive approach to the present. In everyday life, they are more likely to comfort themselves in response to both positive and negative experiences and to encode personal events in a more positive and integrated manner (Leary et al. 2007; Bian 2019). Notably, the high self‐compassion group appeared capable of processing negative memories without excessive avoidance or rumination given that specific autobiographical memories are strongly associated with positive psychological functions—such as problem solving, emotional regulation, and self‐related cognitive integration (Williams et al. 2007). These findings suggest that self‐compassion may facilitate more effective emotional and psychological regulation in the face of stress or failure by optimizing the retrieval of autobiographical memories. Experiments 1 and 2 did not yield fully consistent results with respect to memory vividness. This discrepancy may be attributed to differences in sensitivity between behavioral measures and neural indices. Behavioral ratings rely more heavily on subjective reports, whereas ERP measures are primarily intended to capture the temporal dynamics of cognitive and emotional processing.

The low‐scoring group experienced early suppression of negative memories (N1 amplitude suppression), leading to excessive consumption of cognitive resources and an inability to maintain detailed processing of memory details in the later stages (LPP amplitude reduction). This may also be due to the premature termination of deep processing of negative memories to avoid emotional distress, manifested as a rapid decline in LPP late‐stage amplitude. The high‐scoring group demonstrated stable LPP amplitude between 400 and 700 ms and 1000 and 1500 ms, indicating sustained processing across time windows through dynamic resource allocation. In contrast, the low‐scoring group exhibited an “early suppression–late release” pattern, suggesting short‐term efficacy of their emotional regulation strategies. This finding provides direct evidence for a dynamic regulation model of self‐compassion.

The results of brain functional connectivity also support this view. During autobiographical memory retrieval, the high self‐compassion group exhibited reduced collaborative activity between the central posterior gyrus, precuneus, and other brain regions, consistent with previous studies (Liu et al. 2023). Previous studies on human negative experiences have found that activity in the precuneus is primarily associated with autobiographical memory retrieval (Cavanna and Trimble 2006). Higher self‐compassion may help individuals reduce rumination or reflection on memories, enabling them to detach from negative emotions and prevent excessive dwelling on past experiences. Given the association between the anterior cingulate cortex, negative vocabulary, and rumination (Zhou et al. 2020), the reduced connectivity between the anterior cingulate cortex and other brain regions in the high self‐compassion group may support the ability of self‐compassionate individuals to separate themselves from negative emotions and memories (Liu et al. 2022; Williams et al. 2020). When processing negative self‐related information, self‐compassion reduces experiential self‐immersion and/or past‐focused reflection, helping to reduce the experience of negative emotions (Stawarczyk et al. 2018).

### 4.2 Mechanisms by Which Self‐Compassion Influences Autobiographical Memory Retrieval

From a theoretical perspective, the differences between the high and low self‐compassion groups in the autobiographical memory retrieval process can be explained by integration theory, which is the result of the interaction between the capture/contemplation hypothesis, functional avoidance theory, and executive function impairment hypothesis (Williams 2006). In comparison, the high self‐compassion group engaged in adaptive processing during the early attentional selection phase (N1, 70–130 ms), without experiencing detail suppression caused by emotional avoidance. During the late cognitive integration phase (LPP, 1000–1500 ms), sustained resource allocation via the prefrontal–parietal network (resulting in a larger LPP amplitude) supported the refined reconstruction of memory details. By reducing emotional load and enhancing cognitive control (prefrontal resource allocation), negative memories are processed adaptively, retaining their warning significance (without reducing accessibility) while avoiding rumination (enhancing specificity).

The low self‐compassion group exhibited greater N1 amplitude in response to negative cue words, indicating that they were more easily captured by negative stimuli and influenced by previous schemas. If these stimuli are overprocessed, individuals may become trapped in a level of generalization of self‐related information, leading to the phenomenon of “capture” rumination (Dykas and Cassidy 2011). In the mid‐to‐late stages, compared to the low self‐compassion group, the high self‐compassion group exhibited greater LPP amplitude, indicating their ability to better process past negative autobiographical memories, allocate more cognitive processing resources, and either avoid or become immersed in negative emotions. The low self‐compassion group adopted certain avoidance strategies to evade the emotional distress caused by negative memories, preemptively defending against negative memories to reduce the experience of negative emotions (Wessel et al. 2014).

Brain network analysis further complements the ERP results. From a graph theory perspective, the high self‐compassion group exhibited lower global efficiency and longer feature path lengths in the theta band, consistent with previous literature (Bullmore and Sporns 2012); it should be noted that this finding does not directly indicate superior or inferior processing efficiency. Rather, it may reflect differences in the ways individuals integrate information during self‐referential emotional processing tasks (Bullmore and Sporns 2012; Rubinov and Sporns 2010). Such network characteristics may suggest that individuals with high self‐compassion engage in a more distributed or multi‐pathway mode of information integration when processing negative autobiographical memories, although this interpretation requires further empirical validation. In contrast, the low self‐compassion group may exhibit weaker information modularization at the brain network level. The retrieval of specific autobiographical memories relies heavily on executive control and working memory resources, and deficits in executive functioning may impair the inhibition of irrelevant information as well as the fine‐grained processing of memory details, thereby reducing memory specificity (Williams et al. 2007; Sumner et al. 2011).

In summary, this study systematically explored the impact of self‐compassion on autobiographical memory retrieval, providing a new perspective on how self‐compassion processes negative emotions. It supports the view that self‐compassion is a positive psychological trait that facilitates emotional processing, enhances concrete memory, and activates integrative neural networks. Individuals with high self‐compassion are more accepting of their experiences when faced with difficulties and setbacks due to their high levels of self‐kindness, mindfulness, and sense of common humanity. When recalling memories related to themselves, individuals with high self‐compassion are less susceptible to interference from negative cue words and exhibit lower capture tendencies. They can process memories related to themselves with a more accepting and open attitude, while allocating more cognitive resources to this process. This ability not only helps reduce the impact of negative emotions but may also enhance the specificity and vividness of memory processing, thereby highlighting the unique advantages of self‐compassion in emotional regulation and memory processing. This finding not only provides neurobiological evidence for understanding how self‐compassion promotes mental health but also offers important insights for future emotional regulation training and interventions.

### 4.3 Limitations and Future Directions

This study has several limitations that should be addressed in future research. First, all participants were recruited from a university population, which may limit the generalizability of the findings. Future studies should include more diverse and representative samples across different age groups to enhance the robustness and external validity of the results. Second, emotional variables such as depression and anxiety were not controlled for, and the present design did not distinguish between the encoding and retrieval stages of memory processing. Future research should incorporate these emotional variables and adopt experimental paradigms that directly compare memory encoding and retrieval to further clarify the dynamic mechanisms through which self‐compassion influences autobiographical memory processing. Finally, although EEG provides excellent temporal resolution, its spatial resolution is limited. Future studies could combine EEG with high‐spatial‐resolution neuroimaging techniques, such as functional magnetic resonance imaging (fMRI), as well as other multimodal neuroimaging approaches. In addition, extending the investigation to different types of autobiographical memories and different stages of memory processing would provide a more comprehensive understanding of the neural mechanisms underlying the relationship between self‐compassion and autobiographical memory.

## 5 Conclusions

This study suggests that self‐compassion promotes acceptance and integration of past experiences by regulating individuals' attention allocation during the early stages of autobiographical memory processing and cognitive processing during the middle and late stages. In the future, it may be possible to improve individuals' memory quality and emotional health by enhancing their level of self‐compassion.

## Author Contributions

**Haihong Yin**: data curation, investigation, visualization, Writing – original draft, software. **Xiaozhuang Wang**: conceptualization, writing – review and editing, supervision, methodology. **Wei Hu**: writing – review and editing, validation, formal analysis, supervision, methodology. **Liqing Liu**: writing – review and editing, validation, supervision, methodology. **Yongmei Hu**: project administration, writing – review and editing, funding acquisition.

## Funding

This work was supported by the National Social Science Fund of China (18BTY119 to Yongmei Hu).

## Ethics Statement

The ethics committee of Tianjin Normal University approved this study (No. 2024071206).

## Consent

Informed consent was obtained from all individual participants included in the study.

## Conflicts of Interest

The authors declare no conflicts of interest.

## Data Availability

The data are available from the corresponding author upon reasonable request.

## References

[brb371741-bib-0001] Achard, S. , and E. Bullmore . 2007. “Efficiency and Cost of Economical Brain Functional Networks.” PLoS Computational Biology 3, no. 2: e17. 10.1371/journal.pcbi.0030017. 17274684 PMC1794324

[brb371741-bib-0002] Ahmadi Forooshani, S. , K. Murray , Z. Izadikhah , and N. Khawaja . 2020. “Identifying the Most Effective Strategies for Improving Autobiographical Memory Specificity and Its Implications for Mental Health Problems: A Meta‐Analysis.” Cognitive Therapy and Research 44, no. 2: 258–274. 10.1007/s10608-019-10061-8.

[brb371741-bib-0003] Amodio, D. M. , B. D. Bartholow , and T. A. Ito . 2014. “Tracking the Dynamics of the Social Brain: ERP Approaches for Social Cognitive and Affective Neuroscience.” Social Cognitive and Affective Neuroscience 9, no. 3: 385–393. 10.1093/scan/nst177. 24319116 PMC3980796

[brb371741-bib-0004] Barry, T. J. , D. J. Hallford , and K. Takano . 2021. “Autobiographical Memory Impairments as a Transdiagnostic Feature of Mental Illness: A Meta‐Analytic Review of Investigations Into Autobiographical Memory Specificity and Overgenerality Among People With Psychiatric Diagnoses.” Psychological Bulletin 147, no. 10: 1054–1074. 10.1037/bul0000345. 34968086

[brb371741-bib-0005] Bauer, P. J. , J. Stafford Stevens , F. L. Jackson , and P. San Souci . 2012. “Electrophysiological Indices of Emotion Processing During Retrieval of Autobiographical Memories by School‐Age Children.” Cognitive, Affective, & Behavioral Neuroscience 12, no. 1: 99–114. 10.3758/s13415-011-0073-7. 22135090

[brb371741-bib-0006] Berntsen, D. , and D. C. Rubin . 2008. “The Reappearance Hypothesis Revisited: Recurrent Involuntary Memories After Traumatic Events and in Everyday Life.” Memory & Cognition 36, no. 2: 449–460. 10.3758/MC.36.2.449. 18426073 PMC3044895

[brb371741-bib-0007] Bian, X. H. 2019. “The Impact of Self‐Compassion on Negative Bias and Intervention.” PhD diss., East China Normal University.

[brb371741-bib-0008] Blackie, R. A. , and N. L. Kocovski . 2018. “Forgive and Let Go: Effect of Self‐Compassion on Post‐Event Processing in Social Anxiety.” Mindfulness 9, no. 2: 654–663. 10.1007/s12671-017-0808-9.

[brb371741-bib-0009] Bordier, C. , C. Nicolini , and A. Bifone . 2017. “Graph Analysis and Modularity of Brain Functional Connectivity Networks: Searching for the Optimal Threshold.” Frontiers in Neuroscience 11: 441. 10.3389/fnins.2017.00441. 28824364 PMC5540956

[brb371741-bib-0010] Bullmore, E. , and O. Sporns . 2012. “The Economy of Brain Network Organization.” Nature Reviews Neuroscience 13, no. 5: 336–349. 10.1038/nrn3214. 22498897

[brb371741-bib-0011] Cavanna, A. E. , and M. R. Trimble . 2006. “The Precuneus: A Review of Its Functional Anatomy and Behavioural Correlates.” Brain 129, no. 3: 564–583. 10.1093/brain/awl004. 16399806

[brb371741-bib-0013] Chen, X.‐J. , Y.‐S. Huang , X.‐J. Dang , and X.‐F. Zheng . 2013. “Reduced Specificity of Autobiographical Memory in Traumatized Adolescents: Exploring the Contributions of Impaired Executive Control and Affect Regulation: Reduced Specificity of Autobiographical Memory in Traumatized Adolescents: Exploring the Contributions of Impaired Executive Control and Affect Regulation.” Acta Psychologica Sinica 44, no. 1: 112–120. 10.3724/SP.J.1041.2012.00112.

[brb371741-bib-0014] Conway, M. A. , and C. W. Pleydell‐Pearce . 2000. “The Construction of Autobiographical Memories in the Self‐Memory System.” Psychological Review 107, no. 2: 261–288. 10.1037//0033-295X.107.2.261. 10789197

[brb371741-bib-0015] Creaser, J. L. , J. Storr , and A. Karl . 2022. “Brain Responses to a Self‐Compassion Induction in Trauma Survivors With and Without Post‐Traumatic Stress Disorder.” Frontiers in Psychology 13: 765602. 10.3389/fpsyg.2022.765602. 35391975 PMC8980710

[brb371741-bib-0016] Dai, X. , S. Lu , A. A. Sullivan , and H. Hu . 2024. “All You Need Is Compassion? A Latent Profile Analysis of Neglect and Self‐Compassion on Child Mental Health.” Journal of Affective Disorders 362: 799–807. 10.1016/j.jad.2024.07.096. 39029682

[brb371741-bib-0017] Doan, U. , D. Hong , L. Mares , et al. 2025. “The Predictive Power of Autobiographical Memory in Shaping the Mental Health of Young People: An Individual Participant Data Meta‐Analysis.” Psychological Bulletin 151, no. 4: 455–475. 10.1037/bul0000474. 40310230

[brb371741-bib-0018] Dykas, M. J. , and J. Cassidy . 2011. “Attachment and the Processing of Social Information Across the Life Span: Theory and Evidence.” Psychological Bulletin 137, no. 1: 19–46. 10.1037/a0021367. 21219056

[brb371741-bib-0019] Erdős, P. , and A. Rényi . 1960. “On the Evolution of Random Graphs.” Publications of the Mathematical Institute of the Hungarian Academy of Sciences 5: 17–61.

[brb371741-bib-0020] Faul, F. , E. Erdfelder , A.‐G. Lang , and A. Buchner . 2007. “G*Power 3: A Flexible Statistical Power Analysis Program for the Social, Behavioral, and Biomedical Sciences.” Behavior Research Methods 39, no. 2: 175–191. 10.3758/BF03193146. 17695343

[brb371741-bib-0021] Fields, E. C. , H. J. Bowen , R. T. Daley , K. R. Parisi , A. Gutchess , and E. A. Kensinger . 2021. “An ERP Investigation of Age Differences in the Negativity Bias for Self‐Relevant and Non–Self‐Relevant Stimuli.” Neurobiology of Aging 103: 1–11. 10.1016/j.neurobiolaging.2021.02.009. 33773473 PMC8178198

[brb371741-bib-0022] Foti, D. , G. Hajcak , and J. Dien . 2009. “Differentiating Neural Responses to Emotional Pictures: Evidence From Temporal‐Spatial PCA.” Psychophysiology 46, no. 3: 521–530. 10.1111/j.1469-8986.2009.00796.x. 19496228

[brb371741-bib-0023] Gong, H. , D. Yang , and F. Zhang . 2019. “The Influence of Questioning Type on the Generalization of Autobiographical Memory.” Acta Psychologica Sinica 51, no. 12: 1318–1329. 10.3724/SP.J.1041.2019.01318.

[brb371741-bib-0024] Gong, H. L. , H. L. Jia , T. M. Guo , and L. L. Zou . 2014. “Revision and Psychometric Evaluation of the Adolescent Self‐Compassion Scale.” Psychological Research 7, no. 1: 36‐40+79. CNKI:SUN:OXLY.0.2014‐01‐006.

[brb371741-bib-0072] Gratton, G. , M. G. H. Coles , and E. Donchin . 1983. “A New Method for Off‐line Removal of Ocular Artifact.” Electroencephalography and Clinical Neurophysiology 55, no. 4: 468–484. 10.1016/0013-4694(83)90135-9. 6187540

[brb371741-bib-0025] Hajcak, G. , and T. A. Dennis . 2009. “Brain Potentials During Affective Picture Processing in Children.” Biological Psychology 80, no. 3: 333–338. 10.1016/j.biopsycho.2008.11.006. 19103249 PMC2656402

[brb371741-bib-0026] Hallford, D. J. , D. Rusanov , J. J. E. Yeow , and T. J. Barry . 2021. “Overgeneral and Specific Autobiographical Memory Predict the Course of Depression: An Updated Meta‐Analysis.” Psychological Medicine 51, no. 6: 909–926. 10.1017/S0033291721001343. 33875023

[brb371741-bib-0027] Hiraoka, R. , E. C. Meyer , N. A. Kimbrel , B. B. DeBeer , S. B. Gulliver , and S. B. Morissette . 2015. “Self‐Compassion as a Prospective Predictor of PTSD Symptom Severity Among Trauma‐Exposed U.S. Iraq and Afghanistan War Veterans.” Journal of Traumatic Stress 28, no. 2: 127–133. 10.1002/jts.21995. 25808565 PMC5032642

[brb371741-bib-0028] Huang, Y.‐X. , and Y.‐J. Luo . 2009. “Can Negative Stimuli Always Have the Processing Superiority？: Can Negative Stimuli Always Have the Processing Superiority？.” Acta Psychologica Sinica 41, no. 9: 822–831. 10.3724/SP.J.1041.2009.00822.

[brb371741-bib-0029] Johnson, E. A. , and K. A. O'Brien . 2013. “Self‐Compassion Soothes the Savage EGO‐Threat System: Effects on Negative Affect, Shame, Rumination, and Depressive Symptoms.” Journal of Social and Clinical Psychology 32, no. 9: 939–963. 10.1521/jscp.2013.32.9.939.

[brb371741-bib-0031] Leary, M. R. , E. B. Tate , C. E. Adams , A. Batts Allen , and J. Hancock . 2007. “Self‐Compassion and Reactions to Unpleasant Self‐Relevant Events: The Implications of Treating Oneself Kindly.” Journal of Personality and Social Psychology 92, no. 5: 887–904. 10.1037/0022-3514.92.5.887. 17484611

[brb371741-bib-0032] Leistedt, S. J. J. , N. Coumans , M. Dumont , J. Lanquart , C. J. Stam , and P. Linkowski . 2009. “Altered Sleep Brain Functional Connectivity in Acutely Depressed Patients.” Human Brain Mapping 30, no. 7: 2207–2219. 10.1002/hbm.20662. 18937282 PMC6870637

[brb371741-bib-0033] Li, B. , and X. M. Zhao . 2016. “Reducing the Vividness and Emotional Impact of Autobiographical Memories: The Importance of Self‐Regulation Depletion.” Psychological Science 39, no. 1: 56–62. 10.16719/j.cnki.1671-6981.20160109.

[brb371741-bib-0034] Li, P. , H. Liu , Y. Si , et al. 2019. “EEG Based Emotion Recognition by Combining Functional Connectivity Network and Local Activations.” IEEE Transactions on Biomedical Engineering 66, no. 10: 2869–2881. 10.1109/TBME.2019.2897651. 30735981

[brb371741-bib-0035] Li, Y. , D. Cao , L. Wei , Y. Tang , and J. Wang . 2015. “Abnormal Functional Connectivity of EEG Gamma Band in Patients With Depression During Emotional Face Processing.” Clinical Neurophysiology 126, no. 11: 2078–2089. 10.1016/j.clinph.2014.12.026. 25766267

[brb371741-bib-0036] Liao, K. Y.‐H. , G. B. Stead , and C.‐Y. Liao . 2021. “A Meta‐Analysis of the Relation Between Self‐Compassion and Self‐Efficacy.” Mindfulness 12, no. 8: 1878–1891. 10.1007/s12671-021-01626-4.

[brb371741-bib-0037] Liu, G. , C. Santana‐Gonzalez , T. A. Zeffiro , N. Zhang , M. Engstrom , and K. Quevedo . 2023. “Self‐Compassion and Neural Activity During Self‐Appraisals in Depressed and Healthy Adolescents.” Journal of Affective Disorders 339: 717–724. 10.1016/j.jad.2023.07.012. 37437742

[brb371741-bib-0038] Liu, G. , N. Zhang , J. Y. Teoh , et al. 2022. “Self‐Compassion and Dorsolateral Prefrontal Cortex Activity During Sad Self‐Face Recognition in Depressed Adolescents.” Psychological Medicine 52, no. 5: 864–873. 10.1017/S0033291720002482. 32698918 PMC8208230

[brb371741-bib-0039] Lu, Y. , W.‐N. Zhang , W. Hu , and Y.‐J. Luo . 2011. “Understanding the Subliminal Affective Priming Effect of Facial Stimuli: An ERP Study.” Neuroscience Letters 502, no. 3: 182–185. 10.1016/j.neulet.2011.07.040. 21827830

[brb371741-bib-0040] Luo, X. , X. Che , and H. Li . 2023. “Concurrent TMS‐EEG and EEG Reveal Neuroplastic and Oscillatory Changes Associated With Self‐Compassion and Negative Emotions.” International Journal of Clinical and Health Psychology 23, no. 1: 100343. 10.1016/j.ijchp.2022.100343. 36299492 PMC9577271

[brb371741-bib-0041] Luo, Y. , C. Liu , L. Zheng , and X. Chen . 2020. “Attachment and Autobiographical Memory Retrieval: Event‐Related Potential Evidence From Strategic Information Processing.” Consciousness and Cognition 83: 102980. 10.1016/j.concog.2020.102980. 32645690

[brb371741-bib-0043] Miyagawa, Y. , and J. Taniguchi . 2020. “Self‐Compassion and Time Perception of Past Negative Events.” Mindfulness 11, no. 3: 746–755. 10.1007/s12671-019-01293-6.

[brb371741-bib-0071] Maki, Y. , S. M. J. Janssen , A. Uemiya , and M. Naka . 2013. “The Phenomenology and Temporal Distributions of Autobiographical Memories Elicited with Emotional and Neutral Cue Words.” Memory 21, no. 3: 286–300. 10.1080/09658211.2012.725739. 23215874

[brb371741-bib-0044] Moadab, I. 2013. “The Role of Mindfulness and Self‐Compassion in the Neural Mechanisms of Attention and Self‐Monitoring.” PhD diss., University of Oregon.

[brb371741-bib-0045] Neff, K. 2003. “Self‐Compassion: An Alternative Conceptualization of a Healthy Attitude Toward Oneself.” Self and Identity 2, no. 2: 85–101. 10.1080/15298860309032.

[brb371741-bib-0047] Neff, K. D. , T. A. Whittaker , and A. Karl . 2017. “Examining the Factor Structure of the Self‐Compassion Scale in Four Distinct Populations: Is the Use of a Total Scale Score Justified?.” Journal of Personality Assessment 99, no. 6: 596–607. 10.1080/00223891.2016.1269334. 28140679

[brb371741-bib-0047a] Neff, K. D. 2023. “Self‐compassion: Theory, method, research, and intervention.” Annual Review of Psychology 74: 193218. 10.1146/annurev-psych-032420-031047. 35961039

[brb371741-bib-0048] Pan, J. , L. Zhan , C. Hu , et al. 2018. “Emotion Regulation and Complex Brain Networks: Association Between Expressive Suppression and Efficiency in the Fronto‐Parietal Network and Default‐Mode Network.” Frontiers in Human Neuroscience 12: 70. 10.1080/00223891.2016.1269334. 29662443 PMC5890121

[brb371741-bib-0049] Parrish, M. H. , T. K. Inagaki , K. A. Muscatell , K. E. B. Haltom , M. R. Leary , and N. I. Eisenberger . 2018. “Self‐Compassion and Responses to Negative Social Feedback: The Role of Fronto‐Amygdala Circuit Connectivity.” Self and Identity 17, no. 6: 723–738. 10.1080/15298868.2018.1490344.

[brb371741-bib-0050] Pereira, D. R. , A. Sampaio , and A. P. Pinheiro . 2021. “Interactions of Emotion and Self‐Reference in Source Memory: An ERP Study.” Cognitive, Affective, & Behavioral Neuroscience 21, no. 1: 172–190. 10.3758/s13415-020-00858-6. 33608840

[brb371741-bib-0051] Rubinov, M. , and O. Sporns . 2010. “Complex Network Measures of Brain Connectivity: Uses and Interpretations.” NeuroImage 52, no. 3: 1059–1069. 10.1016/j.neuroimage.2009.10.003. 19819337

[brb371741-bib-0073] Sun, J. , B. Sun , B. Wang , and H. Gong . 2012. “The Processing Bias for Threatening Cues Revealed by Event‐related Potential and Event‐related Oscillation Analyses.” Neuroscience 203: 91–98. 10.1016/j.neuroscience.2011.12.038. 22233779

[brb371741-bib-0052] Shin, N. Y. , and Y. Lim . 2019. “Contribution of Self‐Compassion to Positive Mental Health Among Korean University Students.” International Journal of Psychology 54, no. 6: 800–806. 10.1002/ijop.12527. 30206928

[brb371741-bib-0053] Shiota, S. 2024. Perspective Chapter: Association Among Fantasy, Metacognition, and Autobiographical Memory in Self‐Compassion During Empathy and Its Psycho/Neuro/Biological Basis. IntechOpen. 10.5772/intechopen.1004269.

[brb371741-bib-0054] Stawarczyk, D. , O. Jeunehomme , and A. D'Argembeau . 2018. “Differential Contributions of Default and Dorsal Attention Networks to Remembering Thoughts and External Stimuli From Real‐Life Events.” Cerebral Cortex 28, no. 11: 4023–4035. 10.1093/cercor/bhx270. 29045587

[brb371741-bib-0055] Stutts, L. A. , M. R. Leary , A. S. Zeveney , and A. S. Hufnagle . 2018. “A Longitudinal Analysis of the Relationship Between Self‐Compassion and the Psychological Effects of Perceived Stress.” Self and Identity 17, no. 6: 609–626. 10.1080/15298868.2017.1422537.

[brb371741-bib-0056] Sumner, J. A. , J. W. Griffith , S. Mineka , K. N. Rekart , R. E. Zinbarg , and M. G. Craske . 2011. “Overgeneral Autobiographical Memory and Chronic Interpersonal Stress as Predictors of the Course of Depression in Adolescents.” Cognition and Emotion 25, no. 1: 183–192. 10.1080/02699931003741566. 21432666 PMC3481543

[brb371741-bib-0057] Tanguay, A. F. N. , A.‐K. Johnen , I. Markostamou , et al. 2022. “The ERP Correlates of Self‐Knowledge in Ageing.” Memory & Cognition 50, no. 3: 564–585. 10.3758/s13421-021-01225-7. 34432267 PMC8938391

[brb371741-bib-0057a] Tulving, E. 2002. “Episodic memory: From mind to brain.” Annual Review of Psychology 53: 125. 10.1146/annurev.psych.53.100901.135114. 11752477

[brb371741-bib-0058] Valdez, C. , and M. Lilly . 2015. “Self‐Compassion and Trauma Processing Outcomes Among Victims of Violence.” Mindfulness 7: 329–339. 10.1007/s12671-015-0442-3.

[brb371741-bib-0059] Van Wijk, B. C. , C. J. Stam , and A. Daffertshofer . 2010. “Comparing Brain Networks of Different Size and Connectivity Density Using Graph Theory.” PLoS ONE 5, no. 10: e13701. 21060892 10.1371/journal.pone.0013701 PMC2965659

[brb371741-bib-0061] Wessel, I. , I. R. Postma , R. J. C. Huntjens , et al. 2014. “Differential Correlates of Autobiographical Memory Specificity to Affective and Self‐Discrepant Cues.” Memory 22, no. 6: 655–668. 10.1080/09658211.2013.811255. 23889508

[brb371741-bib-0062] Williams, J. M. , and K. Broadbent . 1986. “Autobiographical Memory in Suicide Attempters.” Journal of Abnormal Psychology 95, no. 2: 144–149. 10.1037/0021-843X.95.2.144. 3711438

[brb371741-bib-0063] Williams, J. M. G. 2006. “Capture and Rumination, Functional Avoidance, and Executive Control (CaRFAX): Three Processes That Underlie Overgeneral Memory.” Cognition and Emotion 20, no. 3–4: 548–568. 10.1080/02699930500450465. 26529222

[brb371741-bib-0064] Williams, J. M. G. , T. Barnhofer , C. Crane , et al. 2007. “Autobiographical Memory Specificity and Emotional Disorder.” Psychological Bulletin 133, no. 1: 122–148. 10.1037/0033-2909.133.1.122. 17201573 PMC2834574

[brb371741-bib-0065] Wilson, D. , E. V. Bennett , A. D. Mosewich , G. E. Faulkner , and P. R. E. Crocker . 2019. ““The Zipper Effect”: Exploring the Interrelationship of Mental Toughness and Self‐Compassion Among Canadian Elite Women Athletes.” Psychology of Sport and Exercise 40: 61–70. 10.1016/j.psychsport.2018.09.006.

[brb371741-bib-0065a] Wenzel, A. , K. Pinna , and D. C. Rubin . 2004. “Autobiographical memories of anxiety‐related experiences.” Behaviour Research and Therapy 42, no. 3: 329341. 10.1016/S0005-7967(03)00142-6. 14975773

[brb371741-bib-0066] Winders, S.‐J. , O. Murphy , K. Looney , and G. O'Reilly . 2020. “Self‐Compassion, Trauma, and Posttraumatic Stress Disorder: A Systematic Review.” Clinical Psychology & Psychotherapy 27, no. 3: 300–329. 10.1002/cpp.2429. 31986553

[brb371741-bib-0074] Williams, K. , R. Elliott , S. McKie , R. Zahn , T. Barnhofer , and I. M. Anderson . 2020. “Changes in The Neural Correlates of Self‐Blame Following Mindfulness‐Based Cognitive Therapy in Remitted Depressed Participants.” Psychiatry Research: Neuroimaging 304: 111152. 10.1016/j.pscychresns.2020.111152. 32717664

[brb371741-bib-0066a] Yang, Z. L. , L. P. Guo , P. Wang , et al. 1999. Memory psychology (2nd ed.). East China Normal University Press. [in Chinese].

[brb371741-bib-0067] Zangri, R. M. , P. Roca , I. Blanco , S. Baliyan , and C. Vázquez . 2025. “The Power of Negative Autobiographical Memories: Regulating Affect Through Intensive Mindfulness and Compassion Interventions in a Crossover Design.” Cognitive Therapy and Research. 10.1007/s10608-025-10662-6.

[brb371741-bib-0068] Zhang, H. Y. , and Y. Xu . 2011. “Standardization of Cue Words in the Autobiographical Memory Test (AMT).” Psychological Exploration 6: 503–507.

[brb371741-bib-0069] Zhang, J. , J. Wang , Q. Wu , et al. 2011. “Disrupted Brain Connectivity Networks in Drug‐Naive, First‐Episode Major Depressive Disorder.” Biological Psychiatry 70, no. 4: 334–342. 10.1016/j.biopsych.2011.05.018. 21791259

[brb371741-bib-0070] Zhou, H.‐X. , X. Chen , Y.‐Q. Shen , et al. 2020. “Rumination and the Default Mode Network: Meta‐Analysis of Brain Imaging Studies and Implications for Depression.” NeuroImage 206: 116287. 10.1016/j.neuroimage.2019.116287. 31655111

